# Anthracycline induced cardiotoxicity: biomarkers and “Omics” technology in the era of patient specific care

**DOI:** 10.1186/s40169-017-0148-3

**Published:** 2017-05-10

**Authors:** Shayan Moazeni, Martin Cadeiras, Eric H. Yang, Mario C. Deng, Kim-Lien Nguyen

**Affiliations:** 1 Division of Cardiology, VA Greater Los Angeles Healthcare System, Los Angeles, CA USA; 2 Division of Cardiology, Department of Medicine, David Geffen School of Medicine at UCLA, 11301 Wilshire Blvd, MC 111E, Los Angeles, CA 90024 USA; 3 Division of Molecular Medicine, Department of Anesthesiology, David Geffen School of Medicine at UCLA, Los Angeles, CA USA

**Keywords:** Anthracycline, Biomarkers, Cardiotoxicity, Cardiomyopathy, Heart failure, Omics

## Abstract

Anthracyclines are highly effective against a variety of malignancies. However, their dose-dependent cardiotoxic effects can potentially limit their use. In the past decade, serum biomarkers have been used to diagnose, monitor, predict, and prognosticate disease. Biomarkers such as cardiac troponin and natriuretic peptides have some predictive value, but still lack reliability in this patient population. Novel biomarkers such as galectin-3, soluble ST-2 proteins, myeloperoxidase, and fibrocytes are being explored as potential biomarkers to reliably predict the onset of cardiotoxicity. Leveraging multiomics technology to map highly sensitive biomarkers in an integrated approach through pattern deconvolution may better define those at highest risk of developing cardiotoxicity and further the goal of precision medicine. In this work, we aim to provide a brief overview of traditional serum biomarkers, summarize current investigations on novel circulating biomarkers, and discuss a systems-based approach to anthracycline-induced cardiotoxicity through “omics” technology.

## Introduction

**Fig. 1:**
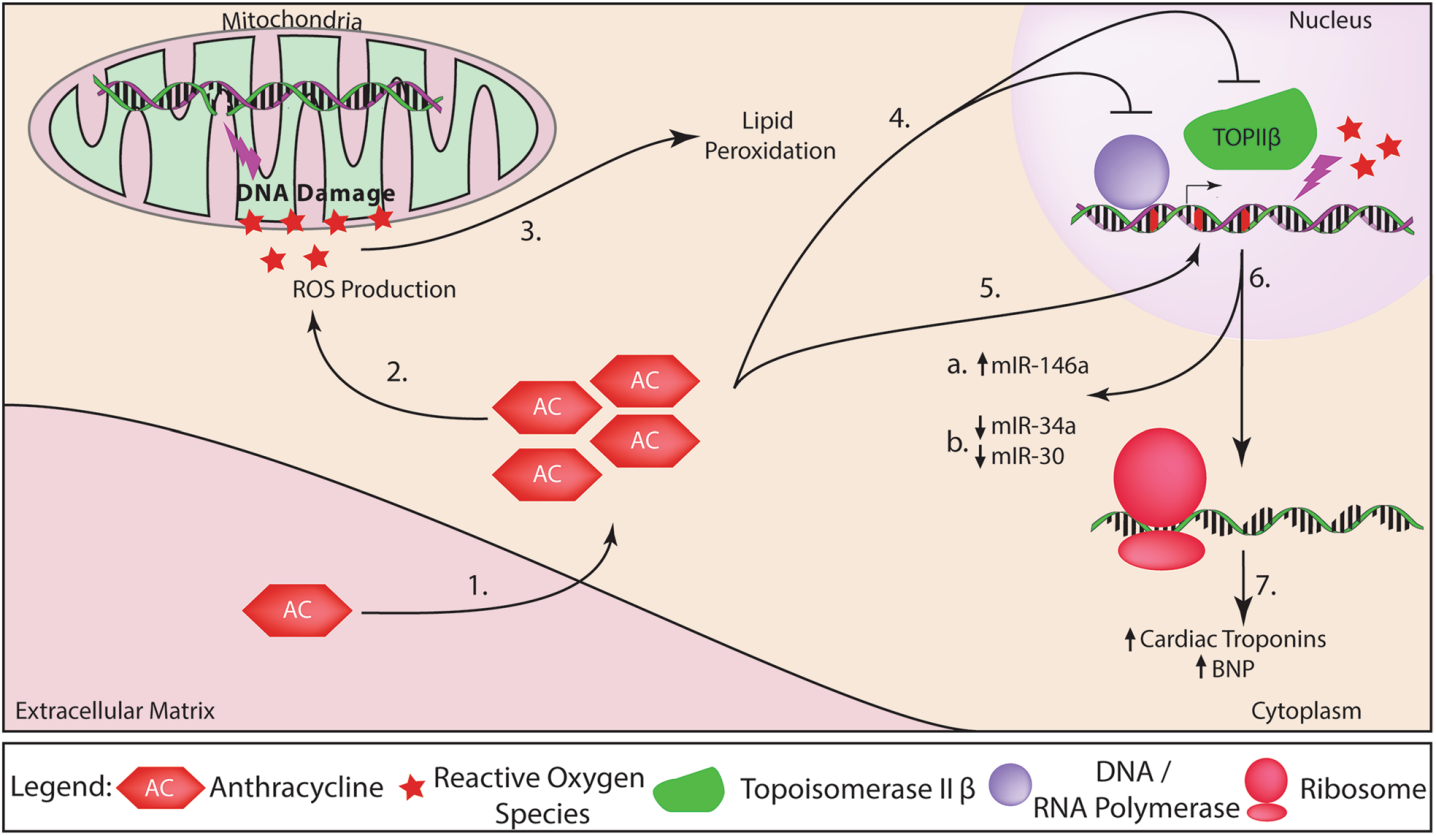
Actions of anthracyclines within the cell. Anthracyclines (ACs) enter the cell, cause mitochondrial damage, and impair transcription in nuclear DNA. *1* In healthy cells, ACs enter the cell’s cytoplasm and has cytotoxic effects. *2* Products from AC-induced ROS (reactive oxygen species) damage mitochondrial DNA leading to mitochondrial dysfunction. *3* ROS-induced generation of oxidized lipid further damages the host‘s cells. *4* AC also inhibits DNA/RNA synthesis by impairing the initiation or elongation phases during DNA synthesis and blocking transcription factor binding or RNA polymerase activity during RNA synthesis. Additionally, intercalation of AC within the DNA can inhibit Topoisomerase II β (TOP2β) by activating the DNA-damage response that leads to cell death. *5* DNA intercalation further impairs normal DNA/RNA synthesis. *6* ACs can function as transcriptional inhibitors and/or modifiers for translational transcripts: (*a*) miR-146a is a negative regulator for epithelial growth factor receptor 4 (ErbB4) and is upregulated shortly after AC exposure. (*b*) Both miR-30 and miR-34a are direct regulators for cell apoptosis and are downregulated shortly after AC treatment in humans and mice. As a result of cardiac injury, troponins and BNP are upregulated due to cardiomyocyte injury and increased circulating levels are detected. Upregulation can result from activation of pro-apoptotic/anti-proliferative pathways that could ultimately lead to cardiac dysfunction if not treated

Cancer survival rates have increased to 67% for patients living past the age of 65 [1]. As a result, there has been a surge of interest to prevent and mitigate adverse sequelae of cancer therapy. While long-term vascular dysfunction has been described in multiple types of cancer therapeutics, one major class of drugs called anthracyclines (AC) is associated with cardiac dysfunction [2]. ACs are most commonly used to treat both solid and hematologic cancers such as leukemia, lymphomas, and breast carcinomas [3]. However, use of ACs is frequently hindered by its dose-dependent cardiotoxic effects [4–6]. When administered during treatment, AC acts as a DNA transcription/replication inhibitor and can further generate reactive oxygen species (ROS) [3]. One mechanistic hypothesis of anthracycline-induced cardiotoxicity (ACIC) relates to oxidative damage and mitochondrial dysfunction, which leads to cardiomyocyte apoptosis (Fig. 1). Additionally, topoisomerase II β (TOP2β) has been highlighted as a novel target for AC and its expression has been shown to correlate with doxorubicin-induced apoptosis. Intercalated ACs inhibit TOP2β from binding to DNA and prevent TOP2β from acting on DNA during replication and transcription leading to cell death [7].

By convention, cardiotoxicity has been classified based on reversibility (i.e. type I vs type II). Type I is irreversible cellular damage; type II is reversible and typically refers to damage related to trastuzumab (an agent sometimes used in conjunction with ACs). While this definition has some molecular basis, strictly classifying cardiotoxicity by type I vs II is limiting and may not apply to newer therapeutics. Some groups have shown partial recovery in cardiac function when cardioprotective medications were used after ACIC (type I) [8]; they attributed the recovery to early detection. Furthermore, in some cases, persistent cardiac dysfunction in trastuzumab-induced cardiotoxicity (type II) [9, 10] was unexpected.

Another method for classifying cardiotoxicity is by temporal occurrence (i.e. acute vs chronic). Acute cardiotoxicity may occur during initial exposure and may persist up to 24 h after AC administration [2, 11, 12]. In contrast, chronic cardiotoxicity can manifest within a year (early onset) or many years after exposure (late onset) and is likely related to a multiple-hit hypothesis, in which the initial myocardial injury persists and predisposes cancer survivors to a potential lifetime risk of functional decline [2, 12–14]. Lifestyle factors and subsequent development of comorbid conditions may also impair the cardiovascular system’s repair mechanisms and potentiate injury [2, 15–19]. The timeline for chronic cardiotoxicity may be months to years after AC treatment and typically results in dilated cardiomyopathy with progressive heart failure [2, 3, 6]. Patients exposed to a cumulative dose of ≥300 mg/m^2^ of doxorubicin (or its equivalent) are considered high risk for developing ACIC. However, evidence supports myocardial injury even at lower AC doses, particularly when administered concomitantly with mediastinal radiation therapy [2, 3, 6].

In this work, data on traditional serum cardiac biomarkers and their role in predicting ACIC will be reviewed. Novel circulating biomarkers that show promise for the detection and prognostication of subclinical cardiotoxicity in cancer patients will be highlighted and the role of multiomics technology for biomarker development in the era of individualized patient care will be discussed. While technologies such as echocardiography, magnetic resonance imaging, or multi-gated acquisition nuclear scans provide downstream direct visualization of structural damage in ACIC and complement early abnormal levels of serum biomarkers, the primary focus for this paper will be on circulating biomarkers and multiomics technology. The reader may consider other published works for a more thorough discussion on the role of imaging technologies in the diagnosis and monitoring of cardiotoxicity [20].

## Traditional serum biomarkers

Serum biomarkers are biologically relevant to a disease pathway and are used as indicators of disease or predictors of disease onset [21]. In the past decade, biomarkers have been used to (1) predict who will develop cardiotoxicity, (2) identify cardiotoxicity while receiving therapy, (3) identify and quantify non-reversible damage in those who receive therapy, and (4) identify those at risk of imminent or recurrent heart failure. Two widely used and well-validated serum biomarkers, which have been used to detect myocardial injury and ventricular wall stress, include cardiac troponins and natriuretic peptides (BNP), respectively. These biomarkers in addition to their ability to predict ACIC have also shown a correlation with all-cause mortality in patients with various malignancies and no prior anticancer treatment [22]. Table 1 summarizes clinical studies that investigated the role of troponin and natriuretic peptides in AC-induced cardiotoxicity across a wide age range.

**Table 1:** Clinical studies investigating the role of cardiac troponins and pro-brain natriuretic peptide in anthracycline-induced cardiotoxicity

Refs.	Author	Cancer type	N	Age (year)	AC analog	AC dose (mg/m^2^)	Time interval	Cardiac troponins	Pro-BNP
[4]	Ky (2014)	Breast cancer	78	50	Doxorubicin	60	0, 3, 6, 15 m	SE (TnI) Significant after 3 m with a 23.4% predictive value	NSE
[5]	Sawaya (2012)	Breast cancer	81	50 ± 10	Doxorubicin Epirubicin	240 300	0, 3, 6, 15 m	SE (cTnI) Significant in low and high dose groups	NSE
[11]	Broeyer (2008)	Various malignancies	26	46 ± 15	Doxorubicin Variable AC	520	0, 4, 24 h	NSE	SE
[24]	Armenian (2014)	Hematologic malignancies	100 (HR) 50 (LR)	26.6 20.8	Variable AC	<300 >300	>10 year >10 year	NSE	SE
[25]	Cardinale (2004)	Various malignancies	703	47 ± 12	High dose chemotherapy	–	1, 3, 6, 9, 12 m	SE (cTnI)	–
[29]	Dodos (2008)	Hematologic malignancies	100	70	Doxorubicin	>300	24 h, 12 m	NSE (TnI)	NSE
[30]	Feola (2009)	Breast cancer	53	55.3	Doxorubicin	60	0, 1, 2, 3 m	Significant elevation after 1 month followed by steady non-significant decrease (TnI)	SE
[34]	Romano (2011)	Breast cancer	71	18–75	Doxorubicin Epirubicin	300 540	Day 1-6, 3, 6, 12 m	NSE	SE
[48]	Harake (2012)	Hematologic malignancies	100	>18	Doxorubicin	300	0 and 3 m	SE (TnT) High dose	SE High dose
[51]	Putt (2015)	Breast cancer	78	<18	Doxorubicin		0, 3, 6, 15 m	SE (TnI)	NSE
[54]	Oliveira-Carvalho (2015)	Breast cancer	59	–	Doxorubicin	60	0, 3, 6, 9, 12 m	SE (TnI)	–

*AC* anthracyclines, *m* month, *LOE* level of evidence, *NSE* No Significant Elevation of Serum Biomarker and Association with Cardiotoxicity, *SE* Significant Elevation of Serum Biomarker and Association with Cardiotoxicity, *y* years, *HR* high risk, *LR* low risk

### Cardiac troponin

Troponin is a protein found in the contractile units of skeletal, cardiac and smooth muscles. Cardiac troponins, such as troponin-T (TnT) and troponin-I (TnI), are considered highly sensitive and specifically found within the contractile units of cardiomyocytes [6, 23]. These biomarkers have been used as sensitive and specific diagnostic markers for the detection of myocardial infarction or cardiac cell damage and therefore, have also been used to predict acute AC-induced cardiotoxicity.

To date, investigations relating to early onset cardiotoxicity and cardiac troponins represent the bulk of published reports. The definition of cardiotoxicity in both studies was based on the Cardiac Review and Evaluation Committee’s (CREC) criteria of drug-associated cardiotoxicity: a reduction in left ventricular ejection fraction (LVEF) of ≥5 to <55% with symptoms of heart failure (HF) or an asymptomatic reduction of LVEF of ≥10 to <55% [4, 5, 24]. This is due to a strong association observed between high AC dosages (total cumulative dose of doxorubicin and its dose equivalent of ≥300 mg/m^2^) and significant elevation of cardiac troponins in early onset cardiotoxicity [4, 5, 25]. Early increases in cardiac troponins (especially within 72 h of treatment) and persistent elevation can herald subsequent cardiac damage [4–6, 24]. In two studies by Cardinale et al. (n = 703) and Auner et al. (n = 78), a significant change between TnI and TnT was shown, each respectively with a significant drop in LVEF after 1 and 6 months after the start of treatment [26, 27]. While cardiac troponins may be useful indicators of cardiomyocyte injury, there are limitations in their reliability to predict cardiotoxicity, cardiomyopathy, subsequent HF, as well as overall prognosis.

Although it is known that cardiac troponin elevation is frequently associated with high AC dosages, there have been studies demonstrating injury at low-to-moderate AC doses (<300 mg/m^2^) [4, 5]. Using ultra-sensitive cTn assays, Sawaya et and Ky et al. showed that cardiac troponins had significant predictive value in breast cancer patients receiving low-to-moderate AC doses. Sawaya et al. reported a TnI concentration of ≥30 pg/mL in 32% of their patients developing cardiotoxicity while Ky et al. reported a 31.6 to 33.9% probability of patients with elevated cTnI levels. One should note that the patient population in the studies conducted by Sawaya et al. and Ky et al. were exposed to trastuzumab in addition to ACs. Because trastuzumab has been known to cause incremental myocardial injury when used in conjunction with ACs [28, 29], both study populations were at higher risk of cardiotoxicity. The difference in patient population may be one reason why sometimes cardiac troponins were not predictive of ACIC [30, 31]. Based on studies reviewed (Table 1), the conflict in findings may also be attributable to the sensitivity of varying troponin assays. Studies that used ultra-sensitive cTn assays achieved higher precision with diagnostic cutoffs of less than 0.04 ng/mL (cTnI-Ultra assays) [32]. These findings contributed to a recommendation by the American Society of Echocardiography and European Association of Cardiovascular Imaging to check serial troponin I during treatment with agents known to be associated with type I and II cardiotoxicity [20]. They further advised referral to a cardiologist if the results are abnormal.

To date, published work on cardiac troponins have shown efficacy in predicting early onset cardiotoxicity. However, the studies have been unable to find a significant detection of troponin levels late after treatment for the prediction of ACIC [25, 31]. These studies consisted of 150 childhood [25] and 53 adult [31] cancer survivors with hematologic malignancies and breast cancer, respectively. Both reported no detectable elevation of TnT or TnI after a 2-year and 1-year follow-up, respectively. These findings suggest that using cardiac troponins to detect chronic or late-onset cardiotoxicity may be less helpful than its use in the acute setting and that the need for other biomarkers is important for identifying those at greatest risk of developing chronic, undulating myocardial injury with resultant cardiac dysfunction.

### Natriuretic peptides

Natriuretic peptides such as atrial natriuretic peptides (ANP) and brain natriuretic peptides (BNP) are peptide hormones that promote natriuresis to protect the heart from excess mechanical stress or volume overload. BNP has been linked to cardiomyocyte damage after injury [6]. In pressure–volume overload conditions leading to ventricular stretch, expression of BNP is increased and released from the cardiomyocytes of the ventricles. Elevations in BNP and NT-proBNP have been associated with early [4, 6, 11, 30, 33] and late detection [5, 6, 25, 31] of ACIC and can be used as a surrogate for acute myocardial stress.

To date, the published literature shows early predictive association between ACIC and NT-proBNP. In two studies, NT-proBNP levels increased significantly after AC treatment [11, 34]. In one study consisting of 21 patients (age 46 ± 15 years) with various malignancies treated with doxorubicin therapy (520 mg/m^2^), detectible NT-proBNP levels were observed as early as 4 h after the start of therapy (18% increase from baseline, p > 0.01); these levels remained persistently elevated after 24 h (238% increase from baseline) [11]. Romano et al. also showed that NT-proBNP was a good predictive marker for subsequent cardiotoxicity in patients who had persistent elevations as early as 3 months after the initial dose of therapy [34]. Cardiotoxicity in this case, was defined as a decrease in LVEF of ≥20% and/or an increase in LV end systolic volume of ≥15% from baseline. In this study, seventy-one breast cancer patients received 6 cycles of liposome-encapsulated doxorubicin (40–50 mg/m^2^), docetaxel (50 mg/m^2^), and epirubicin (90 mg/m^2^) in combination with fluorouracil and cyclophosphamide. At the completion of treatment, NT-proBNP levels were 277% higher than healthy controls. Of greater significance however, is that early NT-proBNP elevations (24 h) were associated with a sequential drop in LVEF. These increases in NT-proBNP returned to baseline after each cycle of AC. The transient increases in NT-proBNP seen with AC exposure in the setting of relatively stable LVEF suggest that there may be some degree of reversibility within the repair mechanism of cardiac homeostasis or that there may be a threshold effect. However, prolonged exposure to AC and/or combination therapy can lead to worsening cardiac reserve and has been shown in a study that detected significant dose-dependent changes in LV function between two groups receiving different cumulative doses of AC (high risk group: ≥300 mg/m^2^ and low risk group: <300 mg/m^2^) [25].

While many studies have evaluated the role of NT-proBNP in detecting early-onset of ACIC, late-onset ACIC has also been associated with increased NT-proBNP levels. Feola et al. reported persistent elevations for up to 2 years after AC treatment, which was associated with an LVEF decline of ≥10% [31]. These findings may suggest long-term neurohormonal activation, which may influence natriuresis, increased sympathetic activity, and increased blood vessel tone and could represent one underlying mechanism for late onset cardiotoxicity [6, 31, 34]. However, because of BNP’s lack of sensitivity in predicting ACIC [4], combining conventional biomarkers with newer biomarkers that have demonstrated stronger potential to predict heart failure or other cardiac related events may improve the detection of cardiotoxicity prior to a decline in LVEF.

## Novel circulating biomarkers

Unlike the traditional biomarkers described above, newer biomarkers have not been fully incorporated into daily clinical use. While there are many promising circulating biomarkers including PlGF, GDF-15, sFlt1, hs-CRP, glycogen phosphorylase BB, and H-FABP, which may give additional insight on the role of growth, proliferation, and/or inflammation in heart failure, we will focus our attention on galectin-3, soluble ST-2, myeloperoxidase, and fibrocytes [6]. Of the novel biomarkers below, serum galectin-3 has been adopted by some academic institutions during routine clinical evaluation of heart failure. Table 2 summarizes both pre-clinical and clinical studies that have assessed the role of novel serum biomarkers in AC-induced cardiotoxicity.

**Table 2:** Studies investigating novel biomarkers for anthracycline-induced cardiotoxicity

Refs.	Author	Cancer type	N	Model	Age (year)	AC analog	AC dosage	Interval time	Galectin-3	sST2	MPO	miRNA
[4]	Ky (2014)	Breast cancer	78	Human	50	Doxorubicin	60 mg/m^2^	0, 3, 6, 15 m	NSE^a^	–	SE^a^	–
[5]	Sawaya (2012)	Breast cancer	81	Human	50 ± 10	Doxorubicin Epirubicin	240 mg/m^2^ 300 mg/m^2^	0, 3, 6, 15 m	–	NSE^b^	–	–
[24]	Armenian (2014)	Hematologic malignancies	100 (HR) 50 (LR)	Human	13.1 6.1	Variable AC	<300 mg/m^2^ >300 mg/m^2^	>10 year >10 year	NSE	NSE	–	–
[30]	Feola (2009)	Breast cancer	53	Human	55.3	Doxorubicin	60 mg/m^2^	0, 1, 2, 3 m	NSE	NSE	–	–
[51]	Putt (2015)	Breast cancer	78	Human	48	Doxorubicin	78 mg/m^2^	0, 3, 6, 15 m	NSE	–	SE	–
[54]	Oliveira-Carvalho (2015)	Breast cancer	59	Human	>18	Doxorubicin	60 mg/m^2^	0, 3, 6, 9, 12 m	–	–	–	NSE (miR-208a) Low dose AC
[55]	Horie (2010)	–	8	Mice	>18	Doxorubicin	20 mg/kg	0, 6, 12, 24 h	–	–	–	SE (miR-146a)
[56]	Desai (2014)	–	12	Mice	>18	Doxorubicin	3–24 mg/kg	2, 3, 4, 6 weeks	–	–	–	SE (miR-34a)
[57]	Roca-Alonso (2015)	Breast cancer	5	Mice	Adult	Doxorubicin	15 mg/kg	0, 5 weeks	–	–	–	SE (miR-30)

*AC* anthracyclines, *h* hours, *m* month, *MPO* myeloperoxidase, *NSE* No Significant Elevation of Serum Biomarker and Association with Cardiotoxicity, *SE* Significant Elevation of Serum Biomarker and Association with Cardiotoxicity, *sST*-*2* soluble ST-2, *y* years, *HR* high risk, *LR* low risk

^a^Baseline measurement of biomarker is above normal

^b^Increased predictability detected when analyzed with cTnI

### Galectin-3

Recently, galectin-3 (gal3) has been considered as a potential biomarker for predicting early or late onset of cardiotoxicity. Gal3 is a chimaera-type galectin that is currently used as a biomarker for damage and development of myocardial fibrosis in heart failure [35, 36]. To determine if gal3 has early predictive value for breast cancer patients, Ky et al. [4] obtained blood samples before and after treatment with doxorubicin, taxanes and/or trastuzumab with periodic follow-up at 3, 6, 9, 12, 15 months from the first day of treatment. Increases of in gal3 were found to be insignificant (p = 0.195) and not predictive of ACIC as defined by echo-derived LVEF reduction. Gal3 was also tested for its ability to predict late-onset ACIC (>10 years from AC exposure date) in a cross-sectional study of AC-exposed childhood cancer survivors [31]. Although survivors treated with AC >300 mg/m^2^ were found to have lower LV wall thickness-to-dimension ratios, increased LV wall stress, and higher myocardial performance index (a marker of poor global systolic and diastolic function), there was no correlation between gal3 and echo-derived cardiac structural changes. Compared to other serum biomarkers, few studies have examined the role of gal3 to detect ACIC. To date, the role of gal3 has not been used to evaluate dose-dependent cardiotoxicity in a wide range of AC dosages. However, because gal3 serves as a surrogate for myocardial fibrosis, it may be possible that more sensitive imaging tools that can quantify fibrosis (such as cardiac magnetic resonance imaging) can show better correlation.

### Soluble ST-2 protein

Soluble suppression of tumorigenicity-2 (sST2) is part of the interleukin 1 receptor family and has been used as a marker for acute myocardial infarction and heart failure [37, 38]. Cardiac hypertrophy, fibrosis, and ventricular dysfunction have been associated with abnormal levels of sST2 [37, 38]. In a study consisting of 145 patients, those with elevated sST2 proteins were at a fourfold increased risk for the onset of heart failure [37].

Current research on sST2 proteins, much like Gal3, is sparse in the area of cardiotoxicity. Sawaya et al. [5] and Armenian et al. [25] both evaluated the predictive role of sST2 proteins for early (3–6 months from the start of treatment) and late (2 years after treatment) cardiotoxicity. Both studies showed that baseline measurements of sST2 were higher in the cancer survivor groups compared to normal average serum concentrations [5, 24, 25]. In the study by Sawaya et al. [5], breast cancer patients receiving 240 mg/m^2^ of doxorubicin or 300 mg/m^2^ of epirubicin had baseline pre-treatment sST2 concentrations which were 1.6 times greater than normal serum reference concentrations [5, 39]. This finding suggests that patients may have already been at risk for development of cardiovascular complications before AC treatment or that there may be unknown direct or indirect tumorigenic effects on the myocytes. Similar to cardiac troponins, the longitudinal incremental change in sST2 rather than the absolute value may be predictive but further work is needed.

### Myeloperoxidase

Due to the immediate myocardial damage from AC-induced release of ROS, other potential biomarkers that induce or are a part of the inflammatory response have been under active investigation. One such circulating biomarker is myeloperoxidase (MPO), a pro-inflammatory enzyme expressed by neutrophils, is an indicator of oxidative stress and is involved in lipid peroxidation [40–43]. In one study [44], seventy-eight breast cancer patients were given doxorubicin and trastuzumab (doxorubicin: months 0–3; trastuzumab: months 3–15) and were evaluated over a 15-month period. Measurements of MPO were obtained at the start of treatment and 3-month intervals over the course of 15 months. Cardiotoxicity was determined by echo-derived LVEF reduction using criteria set forth by CREC. Early increases in MPO followed by a sequential decrease were associated with LVEF decline. Also, MPO was proposed as a biomarker for predicting future cardiovascular events such as ACIC in the setting of trastuzumab exposure and heart failure. The authors found that an interval change in MPO levels in the 90th percentile from baseline (2.7-fold increase) carried a 34% probability of developing cardiotoxicity. At 15 months, the risk of future cardiotoxicity was amplified at higher standard deviations. When combined with a TnI elevation in the 90th percentile, the risk of cardiotoxicity at 15 months was 46.5% [4, 6]. These findings suggest that MPO and TnI are highly sensitive biomarkers for improved prognostication of ACIC. However, due to the modest sample size, further assessment of MPO and TnI is needed. Moreover, while increased MPO is associated with ACIC, MPO is also a marker for oxidative stress, and thus may not be specific for ACIC.

### Fibrocytes

Fibrocytes, which are mesenchymal progenitor cells involved in the tissue’s response to injury, have recently been explored as a viable fibrosis biomarker in chronic lung disease, pulmonary hypertension, pulmonary fibrosis, and cardiac diseases such as hypertrophic cardiomyopathy [45, 46]. These cells secrete pro-inflammatory cytokines as well as express chemokine receptors that help facilitate the recruitment of other inflammatory cells in response to tissue injury [47, 48]. Such injuries include the presence of free radicals induced by ROS and chronic cellular apoptosis due to AC treatment, which may be, an important underlying mechanism leading to diffuse myocardial fibrosis. While ionizing radiation causes premature differentiation of fibroblasts to post-mitotic fibrocytes, which actively affect collagen deposition, and while the presence of diffuse myocardial fibrosis in ACIC have been demonstrated using magnetic resonance imaging [49–51], no studies to date however, have specifically correlated levels of fibrocytes with the presence of diffuse myocardial fibrosis secondary to ACIC alone or a combination of ACIC and radiation therapy. One contributing barrier may be that specific flow cytometry [46] thresholds used for quantification of fibrocytes in the setting of ACIC have not been widely validated. Additional investigations are needed to evaluate the role of fibrocytes in ACIC and whether early AC-induced effects on fibrocytes affect downstream myocardial wound healing.

## Leveraging multiomics technology

**Fig. 2:**
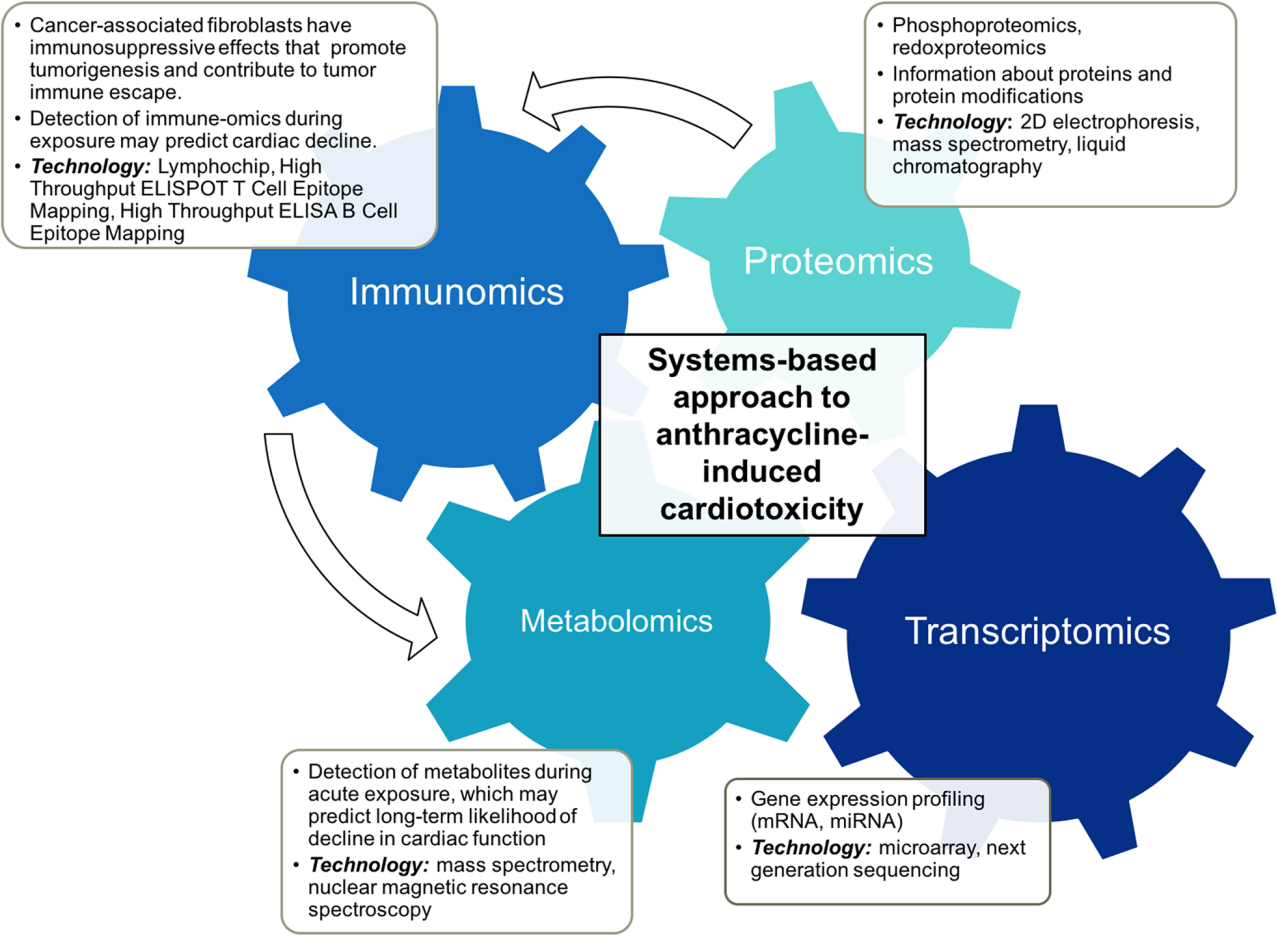
A systems-based approach to understanding anthracycline-induced cardiotoxicity may consist of leveraging –omics technology to identify genomic transcripts (*transcriptomics*), proteins and protein modifications (*proteomics*), and metabolites (*metabolomics*) during acute treatment. Immune-omics may also be leveraged to study the role of immune interactions, which may alter cardiotoxic response. This panoply of data can be coupled with periodic longer-term imaging (based on exposure risk) to further identify those at the greatest risk of developing cardiac dysfunction

With precision medicine as the ultimate goal, multiomics technology can be leveraged to better understand off-targets of ACIC and represents an area ripe for further research and development (Fig. 2). Transcriptomics, proteomics (including phosphoproteomics and redoxproteomics), metabolomics, and more nascent immune-omics can provide an inventory of data relating to changes in cellular levels of mRNA, proteins, protein modifications, metabolites, and immune activation during acute exposure. The ability to look from multiple vistas allows for a systems-biological insight into pathways of acute injury, some of which may contribute to adverse long-term systemic implications. In general, transcriptomics relies on high throughput analysis of genomic transcripts using microarray technology and next-generation sequencing whereas proteomics relies on 2D electrophoresis, mass spectrometry, and liquid chromatography. Metabolomics enable detection of low molecular weight metabolites (acetate, succinate, pyruvate, etc.) by a combination of mass spectrometry and/or NMR (nuclear magnetic resonance) spectroscopy. The bulk of these studies to date are conducted in animal models exposed to ACs, particularly because human tissue specimens (cardiomyocytes) are difficult to obtain. More recent technology has enabled analysis using peripheral leukocytes for gene expression profiling (transcriptomics). Of the –omics technology, transcriptomics and metabolomics have been the ones that seed the path to clinical applications and implementation.

Early pre-clinical work including investigations of miRNA in vitro and in vivo in mice models have shown promise and have triggered interest for patients with ACIC. MiRNA are short non-coding, regulatory RNA strands that regulate post-transcriptional translation of thousands of genes and modulate mRNA stability. These small molecules exhibit a mode of regulating protein expression that is evolutionarily conserved across most vertebrate (human, mice, and etc.) [52] and are involved in cellular physiologic and pathophysiologic processes. MiRNA is detected and quantified using Reverse Transcriptase-Polymerase Chain Reaction (RT-PCR) or sequencing techniques either directly from the tissue of interest such as myocardium or circulating leukocytes but are also found in the circulation. Upon AC administration, changes in miRNA concentration are tested at multiple intervals over a period and quantified based on changes seen across downstream effectors.

Researchers recently proposed that circulating miR-208a (a heart-specific molecule) might be used as an indicator for early drug-induced cardiotoxicity and biomarker of myocardial injury [53]. One study assessed circulating miR-208a expression in 59 female breast cancer patients treated with AC, but were unable to demonstrate associations at 3, 6, 9, and 12 weeks from the first AC dose [54]. Patients received doxorubicin (60 mg/m^2^), cyclophosphamide (600 mg/m^2^) and paclitaxel (80 mg/m^2^) or docetaxel (75 mg/m^2^). Plasma miR-208a was collected alongside serum cTnI in order to compare patients who developed cardiotoxicity vs those who did not. Seven patients developed cardiotoxicity as evidenced by elevation in serum cTnI and decreased in LVEF by echocardiography. MiR-208a was not detected in the bloodstream of the seven breast cancer patients who developed DOX-induced cardiotoxicity. Although the authors concluded that miR-208a is not a good biomarker for DOX-induced cardiotoxicity in breast cancer patients, the number of patients with DOX-induced cardiotoxicity was small. While invasive and challenging in human studies, endomyocardial biopsies of the seven patients with DOX-induced cardiotoxicity for myocardial expression of miR-208a and for proteomics analysis may yield additional insight. Notable in this study was that a fairly low doxorubicin dose was sufficient to induce LVEF decline in seven subjects; this suggests that there may be other patient-specific factors. Studies in larger patient populations are needed before definitive conclusions can be reached.

In mice models, changes in levels of miR-146a, miR-34a, and miR-30 at both low and high concentrations of AC [52, 55–57] have been studied. Horie et al. [55] conducted a study evaluating miR-146a in mice. They showed that miR-146a was upregulated with a subsequent decrease in epithelial growth factor receptor 4 (ErbB4) expression soon after AC treatment. ErbBR represents a family of tyrosine kinase receptors that regulate a variety of cell-specific functions such as differentiation, proliferation, and cell migration. ErbB4 and ErbB2 are both expressed in cardiomyocytes. However, the study was unable to find any significant changes in ErbB2 concentration. On the other hand, the study does suggest that miRNA has a role in detecting cardiotoxicity as some of the mice with upregulated miRNA-146a and downregulated ErbB4 expression developed dilated cardiomyopathy. This type of cardiomyopathy would be fitting due to the stress exhibited in the ventricles of the heart [6, 33, 34, 55].

In recent years, Desai [56] and Roca-Alonso et al. [57] have expanded on miRNA associated with ACIC. They showed detectable changes in the concentration of miR-34a and miR-30 concentration in AC-treated mice. MiR-34a is a direct regulator of apoptosis, which has been linked to the aging heart and is also a marker for myocardial infarction, cardiomyopathy, and heart failure in both human and mice models [56]. Similar to miR-34a, miR-30 downregulation affects the pro-apoptotic BNIP3L/NIC gene, which promotes apoptosis [57]. AC-induced changes in miR-34a and miR-30 concentrations reflect cell death and could mechanistically explain one aspect of the fibrosis associated with AC-induced cardiomyopathy. Additional targets of miR-30 include activation of the β-adrenergic pathway (specifically β1AR and β2AR). Sympathetic activation by the β-adrenergic pathway is also a common effect seen in acute toxicity as a defense mechanism for repair and maintenance of cardiac function. This is another potential area for investigation with natriuretic peptides, which are involved in the maintenance of cardiac function through mediation of sympathetic response [6, 34, 52, 57].

Using gene expression profiling, other groups have used human induced pluripotent stem cell-derived cardiomyocytes (hiPSC-CMs) that have been treated with doxorubicin, daunorubicin, and mitoxantrone to identify 35 common genes involved in the regulation of sarcomeric structures, ion homeostasis, and cell apoptosis [58]. Based on these findings, biomarkers implicated in heart failure (GDF-15 and *GPNMB)* and stress (BAX and FAS) were discovered. For example, growth differentiation factor 15 (GDF-15) has been shown to have predictive value in ACIC where trastuzumab was also implicated [44, 58].

More recently, several studies have also leveraged metabolomics technology in murine models of ACIC. In a study by Li et al., ACIC was induced by doxorubicin, isoproterenol, and 5-fluorouracil [59]. Plasma metabolomics data showed highly specific biomarkers for ACIC including l-Carnitine, 19-hydroxydeoxycorticosterone, lysophosphatidylcholine (LPC; 14:0), and LPC (20:2). Using a vector machine prediction model, incorporation of these metabolites yielded a prediction rate as high as 90%. Other investigators also evaluated metabolites associated with DOX exposure in male B6C3F1 mice using doxorubicin or saline [60]. After DOX 6 mg kg^−1^, there was an increase in cardiac tissue-specific levels of acetylornithine, kynurenine, putrescine and serotonin. Acetylornithine and hydroxyproline were also significantly increased in plasma.

Others have also used proteomic-profiling techniques to identify immunoglobulin E as a highly reduced protein compared to control blood samples in patients receiving doxorubicin/trastuzumab [61]. They concluded that high baseline immunoglobulin E levels were associated with a significantly lower risk of doxorubicin/trastuzumab-induced cardiac dysfunction. These findings implicate the immune system in doxorubicin-induced cardiotoxicity, an angle that has not been considered in cardio-oncology and warrants thorough investigation since immune activation and regulation play a vital role in myocardial homeostasis [62].

In summary, findings from gene expression profiling, proteomics, and metabolomics provide a number of different areas for future research whereas immune-omics are at the cusp of development. Additional studies are needed to assess the ability of new proteins, metabolites, and miRNA to predict ACIC in different types of cancers and their relationship to cumulative AC dose and adjuvant therapy. It should be noted that while gene expression has been very well characterized and studied in many areas of research, there is a lack of consensus around the interpretation of gene expression data as it measures changes in mRNA abundance and not the protein. Uncertainty arises due to the transcriptome being the farthest of the three from the phenotype and the number of regulators that can impact the final expression of the gene [63]. However, the proteome is a dynamic reflection of both genes and the environment making it very promising area for biomarker research as proteins are most likely to be affected due to the etiology of the disease and/or response from the disease [63]. Lastly, the metabolome is the final downstream product gene transcription and is the closest to the phenotype. While the metabolome is a great candidate for biomarker research because of high specificity and changes to the metabolome are amplified relative to the proteome and transcriptome, the metabolome has the smallest domain and is the most diverse. This latter aspect poses additional challenges and analyses are complex [63]. Yet, this is an advancing field and so is the field of precision medicine in which multiple tools are developed to improve not only the detection of a disease but to advance early characterization of phenotypes. New imaging techniques, novel sensors, and wearable technologies aided by powerful computational algorithms bring new horizons to improve the contextualization of circulating biomarkers [64–68].

## Conclusions

To date, traditional biomarkers such as cTn and pro-BNP have shown to be useful in predicting the occurrence of ACIC and future cardiovascular events, but there remains a lack of high yield predictability. While there is ongoing effort to evaluate troponin and BNP during AC therapy prospectively, novel circulating biomarkers may have alternative roles in risk stratification and detection of cardiotoxicity. Other biomarkers such as, PlGF, GDF-15, sFlt1, hs-CRP, MPO, glycogen phosphorylase BB, and H-FABP have shown value in areas of growth/proliferation and inflammation, and thus warrant further evaluation in ACIC [6]. Although applicable in other causes of heart failure, work on gal3 and sST2 in ACIC have been sparse. In recent years, “omic” technologies have been employed to accelerate circulating biomarker development. While nascent, omic technologies, have been effective at providing a systems-based approach to understanding heart failure and arrhythmias. Compared to transcriptomics, a more established area of -omics, other areas such as metabolomics, proteomics, and immune-omics are gaining greater traction as high-throughput technology and faster processing power become widely accessible. Because the effects of many cancer therapeutics are systemic, employing a systems-based approach would efficiently facilitate circulating biomarker discovery in ACIC.
